# Bioengineering advances in pancreatic cancer organoids for reproducible tumor microenvironment modeling

**DOI:** 10.1016/j.isci.2026.116487

**Published:** 2026-06-23

**Authors:** Renyu Chen, Bohui Yin, Yaowei Zhang, Junya Peng, Xiaoliang Guo, Wenming Wu

**Affiliations:** 1 Department of General Surgery, Peking Union Medical College Hospital, Chinese Academy of Medical Sciences and Peking Union Medical College, Beijing 100730, China; 2 College of Information Science and Technology, Beijing University of Chemical Technology, Beijing 100029, China; 3 State Key Laboratory of Complex Severe and Rare Diseases, Peking Union Medical College Hospital, Peking Union Medical College and Chinese Academy of Medical Sciences, Beijing 100730, China; 4 School of Mechatronical Engineering, Beijing Institute of Technology, Beijing 100081, China

**Keywords:** biological sciences, bioengineering, cancer

## Abstract

Pancreatic ductal adenocarcinoma (PDAC) remains lethal due to late-stage diagnosis and limited non-surgical treatment options. Its intratumoral heterogeneity and desmoplastic tumor microenvironment (TME) drive invasion, immune escape, and treatment failure. Patient-derived organoids (PDOs) have emerged as efficient model platforms for simulating the TME and preserving tumor heterogeneity and enabling functional testing *in vitro*; however, conventional PDO cultures lack defined and controllable microenvironmental components and often exhibited limited reproducibility, physiological fidelity, and observability. This review synthesizes recent bioengineering advances that upgrade pancreatic cancer PDO platforms across three interconnected dimensions: (1) engineered extracellular matrices and biofabrication for reproducible construction; (2) co-culture, microfluidic, and bioreactor systems for physiological fidelity; (3) imaging AI and biosensor pipelines for quantitative monitoring. We highlight practical design principles and remaining bottlenecks for standardization, scalability, and clinical translation.

## Graphical abstract

**Figure:**
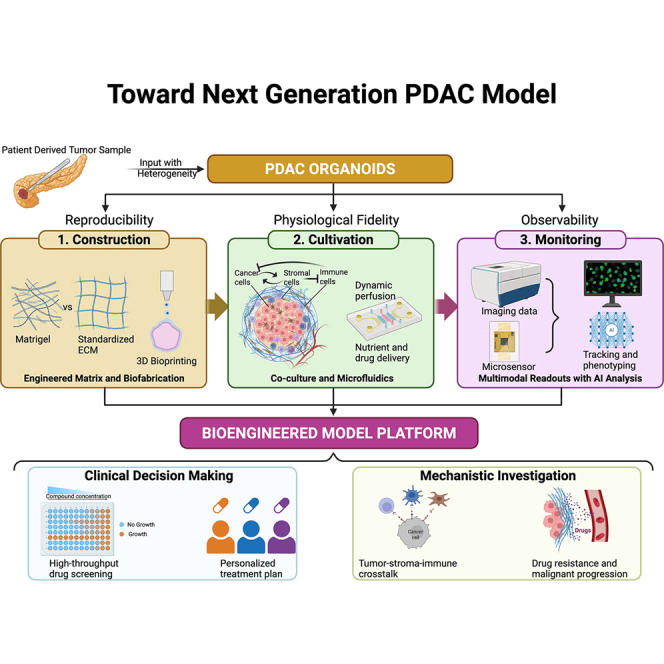

## Introduction

Pancreatic cancer, predominantly **pancreatic ductal adenocarcinoma (PDAC)**, is highly malignant with a 5-year survival rate around 10% and an increasing annual incidence, predicted to become the second leading cause of cancer mortality by 2030.^1^^,^^2^ Despite improvements in diagnostics and treatments, over 50% of patients with PDAC are diagnosed at advanced, unresectable stages, highlighting the urgent need for precise non-surgical therapeutic strategies.^3^ Predicting drug sensitivity in PDAC to guide clinical treatment has become a major focus of research. Therefore, tumor models that replicate the **heterogeneity** and complex microenvironment of primary tumors are fundamental to preclinical studies.^4^ Traditional PDAC research relies heavily on 2D cell lines, 3D tumor spheroids, and mouse models (orthotopic transplantation or genetically engineered mice),^5^^,^^6^ which are not efficient enough to recapitulate the **tumor microenvironment (TME)** in humans. 2D monolayer cultures lack native 3D architecture and ECM cues, 3D spheroids only partially mimic diffusion gradients and are often less patient-specific, and mouse models—while physiologically informative—are typically time-consuming and low throughput for rapid drug sensitivity prediction. Although **patient-derived tumor xenograft (PDTX)** models preserve most of the characteristics of primary tumors, their drawbacks—including the high number of cells required and long proliferation cycles—limit their clinical applicability.^7^ Accordingly, improved models are needed that combine patient-derived tumor features with controllable human-relevant microenvironments (e.g., ECM mechanics, stromal components, and drug gradients) to better mimic the TME and improve functional prediction. In contrast, tumor **organoids**, a 3D culture technology, are widely used in PDAC research to investigate tumor heterogeneity *in vitro*.^8^ The IC50 values for drug sensitivity prediction using PDAC **patient-derived organoids (PDOs)** closely match clinical data, highlighting their role in guiding individualized therapy selection for patients.^9^ Recent single-cell analyses further support the ability of PDAC PDOs to capture multiple malignant cell states and clonal populations, while also indicating that clonal composition can shift with extended culture.^10^ Overall, PDAC PDOs can recapitulate histologic, genetic, and transcriptional characteristics of matched primary tumors and reflect therapy-resistance patterns *in vitro*, supporting their potential for functional drug testing.^11^

**Figure 1:**
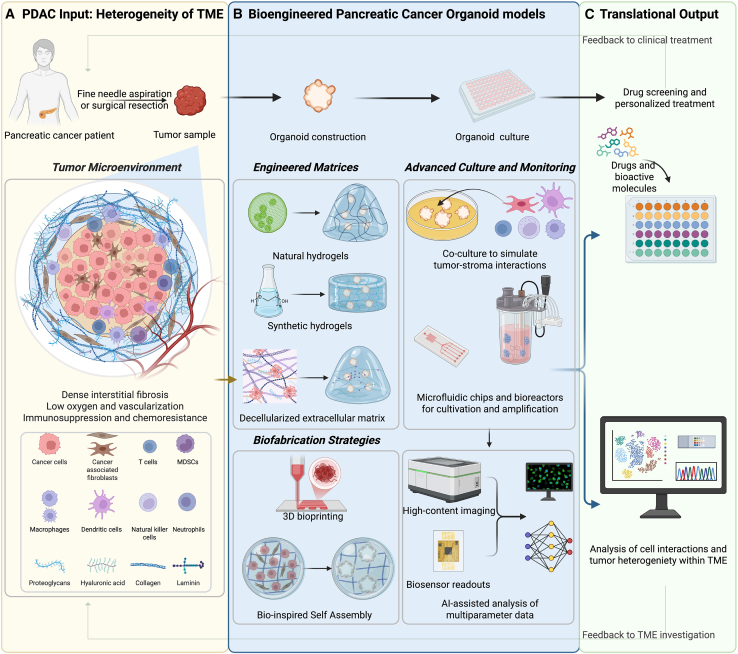
Schematic overview of the bioengineering pipeline for patient-derived pancreatic ductal adenocarcinoma (PDAC) organoids (A) PDAC input: heterogeneity of the tumor microenvironment (TME). Tumor specimens obtained via fine-needle aspiration or surgical resection retain the complex cellular and extracellular components of PDAC, including cancer cells, cancer-associated fibroblasts, immune populations, and a dense fibrotic stroma rich in proteoglycans, hyaluronic acid, collagen, and laminin. (B) Bioengineered organoid models: construction, culture, and monitoring. Patient-derived tumor cells are assembled into three-dimensional organoids within defined microenvironments. **Engineered matrices** encompass natural hydrogels, synthetic hydrogels, and decellularized ECM. **Biofabrication Strategies** utilizing 3D bioprinting and bioinspired self-assembly to balance structural fidelity with throughput. **Advanced culture systems**—co-culture with stromal and immune cells, microfluidic organoid-on-a-chip devices, and dynamic bioreactors—recreate key features of the TME (stromal interactions, vascular perfusion, and shear forces). **Monitoring and analysis** integrate high-content live imaging, AI-driven image segmentation and phenotyping, and real-time biosensor readouts to capture organoid growth, morphology, metabolic state, and drug responses. (C) Translational output. Engineered PDAC organoids feed quantitative multiparametric data into high-throughput drug screening and personalized treatment planning, and furnish mechanistic insights into tumor-stroma-immune crosstalk, thereby closing the loop between basic TME investigation and clinical decision-making. *Created in BioRender*.

Nevertheless, traditional PDO models still have notable limitations. First, they struggle to accurately replicate the TME and reflect interactions between cells and the **extracellular matrix (ECM)**.^12^ Second, they suffer from poor reproducibility throughout preparation, limiting their translation to guiding individualized pharmacological therapy. Recent advancements in bioengineering have enhanced PDO models by improving cell-cell and cell-matrix interactions in the TME and increasing the robustness of organoid production. This offers renewed prospects for advancing PDAC research and treatment using bioengineered PDO models.^13^ Moreover, the FDA’s recent initiative to phase out mandatory animal testing in drug development has shifted momentum toward human-relevant organoid and *in vitro* models. This policy change creates a timely opportunity for a review that highlights cutting-edge organoid technologies. While recent reviews have summarized pancreatic tumor organoid engineering or organoid bioengineering broadly,^12^^,^^13^ a PDAC-focused synthesis that links microenvironment design (matrix and culture), quantitative monitoring/analysis, and translational standardization remains limited. This article reviews recent applications of bioengineering technologies in pancreatic cancer PDO research and provides an integrated “construction-cultivation-monitoring-translation” framework clarifying what each bioengineering strategy adds and what still constrains clinical deployment (Figure 1).

## Matrix engineering for reproducibility

### Beyond matrigel: why standardized matrices are needed

The mechanical properties of ECM are the key to inducing the aggressiveness and drug resistance of PDAC.^14^^,^^15^ Therefore, studying and optimizing the mechanical properties of ECM materials is a key direction in organoid bioengineering.^16^ Traditionally, tumor organoid construction relies on **Matrigel**, an ECM material derived from the ECM of Engelbreth-Holm-Swarm sarcoma and subsequently isolated and purified.^17^ This natural ECM derived from *in vivo* sources is rich in type IV collagen fibers, laminin, heparan sulfate proteoglycans, and various other molecules.^18^ However, because Matrigel is derived from *in vivo* sources, its preparation process is subject to batch effects, making it difficult to ensure stable mechanical properties of the ECM; this in turn affects the reproducibility of organoid experiments and their clinical translational value.^19^ Therefore, there is a need for bioengineering matrices that balance mechanical properties, reproducibility, operability, and material consistency to enhance the potential for foundational and clinical applications of PDAC organoids.

Engineered Matrigel replacements are not merely alternative scaffolds; they act as programmable and tunable microenvironments that decouple matrix mechanics—inherently coupled and poorly defined in Matrigel—thereby enabling causal interrogation of ECM-driven PDAC phenotypes. Current Matrigel replacements for tumor organoids can be organized into three practical classes: (1) natural or purified ECM hydrogels (e.g., collagen I, fibrin, hyaluronan-based gels) that retain native motifs but may still vary in stability and batch consistency^20^^,^^21^^,^^22^^,^^23^; (2) synthetic/semi-synthetic hydrogels (e.g., PEG-based networks) that provide chemically defined, reproducible matrices in which stiffness, stress-relaxation, degradability, and ligand density can be tuned independently^15^^,^^24^^,^^25^^,^^26^^,^^27^; (3) decellularized ECM (tissue- or patient-derived) scaffolds that preserve tissue-specific biochemical complexity but face supply and standardization constraints.^28^^,^^29^ These engineered matrices differ from Matrigel by enabling parameterized control of ECM “design levers,” which is essential for PDAC because desmoplastic mechanics and HA-rich signaling jointly shape tumor plasticity, drug transport, and therapy resistance—phenomena that have been directly demonstrated in PDAC organoids cultured in engineered, stiffness-matched matrices.^13^ Here, we summarize the major categories of extracellular matrices used for tumor organoids and highlight why they are particularly needed for PDAC (Table 1).

**Table 1:** Comparison of bioengineered matrices for PDAC organoids

matrix type	Subtype/example	Key characteristics	Advantages	Limitations	Applications	Ref
Basement membrane extract	matrigel	tumor-derived ECM gel, 4 °C → 37 °C	bioactive, ready-to-use	batch and source variability	gold-standard baseline	^17^^,^^18^^,^^19^
Natural hydrogels	alginate	algae polysaccharide; ion-crosslink	cheap, tunable stiffness	low cell adhesion	brain-metastasis model	^20^
	fibrin (+Laminin-111)	thrombin-crosslinked network	native cell adhesion; tunable stiffness	rapid degradation; batch variability	3D traction assays	^21^
	collagen I	1–2.5 mg/mL conc; stiffness 77–500 Pa	physiologic cues, tunable branching	narrow conc window; morphology sensitive	branching morphometry; ECM stiffness studies	^22^
	collagen I-nanocellulose	blends collagen I with cellulose nanofibres for tunable stiffness (1.2 kPa∼3.3kPa)	increased stiffness than collagen I ECM, co-culture further increases stiffness	potential drug adsorption	stiffness-driven chemoresistance	^30^
	collagen I-matrigel mixture	combines structural scaffolding with biochemical richness	recapitulates PDAC phenotypic plasticity (classical to basal transition), EMT	inherits batch effects from Matrigel	ECM-driven chemoresistance	^31^
	hyaluronic acid	photo-crosslink	viscoelastic, HA-rich TME	needs methacrylation; protease-sensitive; narrow window	desmoplastic niche and macrophage co-culture	^23^
Synthetic hydrogels	PEG	inert, fully defined	highly tunable, bio-orthogonal	needs peptide functionalization	matrix mechanics mimicking	^24^^,^^25^
	GelMA	5% w/v; ∼83% degree of methacrylation; G′ ≈ 6–9 kPa	bioactive; RGD motifs; 3D printable	UV crosslinking; photoinitiator required	bioprinting tumor-scale PDAC stromal models	^26^^,^^27^
	GelNB-CH + HA	Gelatin-based hydrogel crosslinked via norbornene chemistry; tunable elasticity and viscosity; dynamic stiffening from 2 to 6 kPa	mimics PDAC progression by combining dynamic stiffening and HA accumulation; triggers CD44 expression and mesenchymal phenotype; supports CAF invasion	complex fabrication and dual crosslinking; dynamic changes vary across cell types	investigate dynamic stiffening and HA deposition; replicate PDAC TME evolution	^32^
	HA-ELP	HA-benzaldehyde + RGD-ELP-hydrazine; G′≈0.1–4 kPa tunable	fully defined; HA and RGD cues; mimics ECM from soft to tumor-like stiffness	needs recombinant ELP; on-ice mixing	stiffness-driven chemoresistance and HA-CD44 signaling	^15^
	polyacrylamide (PAAm)	synthetic polymer; inert network purely for mechanical control	highly tunable stiffness	not biodegradable; requires functionalization	evaluating matrix rigidity effects	^33^
dECM	patient-derived dECM	native tumor scaffold	preserves TME signals	supply/standardization	Precision-medicine testing	^28^
	Porcine nerve dECM	nerve-specific ECM	perineural invasion model	species differences	PNI mechanism works	^29^
	dSIS-NB	decellularized intestinal mucosa functionalized with norbornene; stiffness 0.9–2.5 kPa	tissue-specific biochemical complexity; tunable stiffness	supply; complex synthesis	differentiation of iPSC-derived pancreatic ductal organoids	^34^
Self-assembling peptides based ECM	PA-E3Y, PA	supramolecular assembly of peptide amphiphiles	highly reproducible, animal-free, easily functionalized	high cost; complex synthesis and optimization	EMT and chemoresistance	^35^^,^^36^

ECM, extracellular matrix; HA, hyaluronic acid; ELP, elastin-like protein; PEG, polyethylene glycol; GelMA, gelatin methacryloyl; dECM, decellularized extracellular matrix; PDO, patient-derived organoid; PNI, perineural invasion; EMT, epithelial-mesenchymal transition; TME, tumor microenvironment.

### Natural hydrogels mimicking physiological conditions

Hydrogels are a class of biocompatible water-absorbing materials that are widely used in the design of drug delivery systems, tissue engineering scaffolds, and wound dressings.^37^ Natural hydrogels are derived from naturally occurring polymers or biomolecules, such as alginate, gelatin, polymerized collagen, and hyaluronic acid methacrylate.^5^^,^^22^^,^^23^^,^^38^ In organoid construction, natural hydrogels can mimic the mechanical and biochemical characteristics of the ECM and are extensively employed in organoid studies to provide structural scaffolding as well as biological cues. For example, although collagen I can be used as a scaffold—either alone or in combination with Matrigel—its baseline stiffness often remains lower than the pathological state.^22^^,^^31^ To address this, when blended with high-stiffness materials, such as nanocellulose, its mechanical rigidity is significantly enhanced to better mimic the desmoplastic PDAC microenvironment. This composite matrix faithfully recapitulates PDAC phenotypic plasticity and epithelial-mesenchymal transition.^30^ Alginates are natural biomaterials extracted from algae, with relatively fewer cell-binding sites. Alginate hydrogel enables superior 3D organoid formation for modeling cancer brain metastasis compared to Matrigel.^20^ Soft fibrin not only exhibits ideal biocompatibility, biodegradability, and tunable mechanical properties but also provides a greater abundance of bioactive binding sites, promoting the growth and functional expression of diverse cell types. Broguiere et al. introduced a defined composite ECM hydrogel using soft fibrin networks supplemented with laminin-111. This matrix provided an animal-free alternative to Matrigel and significantly improved the success rate of organoid initiation from PDAC tissues.^21^

### Synthetic hydrogels for mechanical tunability

While natural hydrogels successfully provide inherent biological cues, their limited mechanical tunability and lack of standardization remain significant challenges. Consequently, synthetic hydrogels have emerged as powerful structural complements that can be integrated with functional natural biomaterials to engineer highly tunable extracellular matrices. They are produced using chemically synthesized polymers such as polyvinyl alcohol, **polyethylene glycol (PEG)**,^39^ and polyacrylamide.^33^ Synthetic hydrogels offer the advantage of stronger and more tunable mechanical properties through adjustments in synthesis conditions and materials, with more consistent and reproducible composition and performance.^21^

Research indicates that four-arm PEG-based hydrogels demonstrate excellent ECM-mimicking properties in 3D culture systems.^24^ Christopher et al. combined self-synthesized and purified polypeptide proteins with adhesive peptides and an eight-arm PEG hydrogel to create multifunctional composite PEG hydrogel materials. This material allows for controlled adjustment of polymer density and matrix characteristics, offering a comprehensive range of mechanical properties that support the growth of mouse and human pancreatic cancer organoids.^25^ Besides, due to their excellent biocompatibility and plasticity, **gelatin methacryloyl (GelMA)**-based polymeric materials are also suitable for use in PDAC organoid systems. The synthesis of GelMA materials involves photopolymerization reactions. Upon the activation of the photocatalyst with visible light, the methacrylate groups facilitate the crosslinking of gelatin molecules and subsequent gel formation.^40^ GelMA-based 3D bioprinting enables the creation of tumor-scale PDAC models, offering a more accurate platform for preclinical drug screening and assessing therapeutic responses.^26^ Meinert’s research team discovered that GelMA hydrogels exhibit mechanical stiffness (1–6 kPa) comparable to the tumor ECM, thereby shortening the duration required for the proliferation and formation of tumor organoids.^27^ Notably, because pure synthetic polymers inherently lack cell-adhesive and signaling motifs, they are routinely functionalized with natural bioactive cues such as HA. Illustrating this hybrid approach, LeSavage et al. developed a hybrid hydrogel combining hyaluronic acid and an elastin-like protein (HA-ELP) to emulate the fibrotic PDAC stroma. Strikingly, organoids in stiff HA-ELP matrices became chemoresistant to gemcitabine, whereas those in softer gels remained drug-sensitive.^15^ In summary, synthetic hydrogels offer more standardized and controllable ECM environments, not only addressing challenges with reproducibility but also helping recapitulate the mechanical properties of the TME, thereby facilitating drug screening. Nevertheless, synthetic matrices typically require deliberate presentation of key biochemical signals (e.g., adhesion ligands and degradability) and may need PDO-specific optimization to achieve robust growth and differentiation across diverse PDAC cultures.

### Decellularized extracellular matrix: preserving native tumor environment

Unlike synthetic hydrogels, **decellularized extracellular matrix (dECM)**, obtained by removing cellular components from tumor tissues or organs, is derived from *in vivo* sources that retain the native fibrous components of the tumor matrix, thus providing a more conducive environment for cell growth.^41^ dECM hydrogels retain multiple cell growth factors, effectively supporting the growth, migration, proliferation, and angiogenesis of stem cells and tumor cells. The formation of dECM hydrogels depends on the self-assembly of collagen fibers. The dECM is freeze-dried into a powder and dissolved in an acidic solution containing proteases for use. By adjusting the solution’s temperature, pH, or adding cross-linking enzymes, dECM can be assembled into a hydrogel with proper rigidity for 3D cell growth.^41^^,^^42^ Patient-derived dECM, due to its fully human-derived nature, effectively simulates the TME and preserves the original tumor structure and ultrastructural features, providing a closer approximation of the actual drug response observed in patients with pancreatic cancer.^28^ Additionally, porcine sciatic nerve dECM hydrogels are used to model PDAC perineural invasion *in vitro*, revealing that PDAC-derived extracellular vesicles promote perineural invasion by activating Schwann cells.^29^

However, a practical limitation is that dECM composition can vary across tissue sources/donors and decellularization protocols, potentially introducing batch-to-batch variability and complicating standardization across studies. To overcome this, recent bioengineering efforts have introduced chemically modified dECM to enhance precise structural control. For instance, decellularized intestinal mucosa functionalized with norbornene (dSIS-NB) introduces photo-crosslinkable properties, while retaining the complex biochemical milieu essential for pancreatic ductal organoid differentiation.^34^

In conclusion, engineered ECMs increase the reproducibility of PDO culture and better recapitulate tumor TME both mechanically and spatially. Notably, proteomic mapping of the PDAC ECM highlights substantial glycoprotein/proteoglycan complexity, including fibronectin- and hyaluronan-associated components, which reshape adhesion programs and rewire invasion and therapy response.^15^^,^^43^ Current engineered matrices remain incomplete surrogates of native PDAC ECM. Not only do they struggle to fully capture its dynamic remodeling and native ingredients, but embedding cells in bulk hydrogels also possesses an inherent spatial limitation: it relies on random cellular aggregation and fails to recreate the precise macroscopic spatial architecture of native pancreatic tumors. To overcome this limitation and reconstruct the spatial heterogeneity of PDAC, advanced biofabrication strategies are required to physically organize these engineered matrices and cells into complex 3D architectures.

## Fabrication strategies balancing reproducibility and biological complexity

### 3D bioprinting: enabling precise placement and shape-forming

Building upon defined matrices, researchers are pursuing automated fabrication methods to standardize production, enhance throughput, and more faithfully recapitulate the spatial architecture of the PDAC microenvironment. Bioprinting turns PDAC organoid culture from a “handcrafted droplet” into a standardized manufacturing process, enabling spatially defined tumor-stroma architectures that can be benchmarked, scaled, and linked to quantitative drug-response phenotypes.^44^ This is particularly relevant for PDAC, where therapy response is strongly shaped by desmoplastic mechanics, HA-rich stromal cues, and spatially organized tumor-stroma interfaces.^45^ From an engineering standpoint, printing modality determines the admissible “bioink window” and the resolution-throughput-viability trade-off. Extrusion-based printing is widely accessible and compatible with viscous, cell-dense bioinks, but it typically sacrifices resolution and exposes cells to nozzle-associated shear, which can impact viability and phenotype.^26^^,^^46^ Droplet/inkjet printing offers higher throughput for arraying microtissues, yet operates within a narrower viscosity range and can limit achievable cell density/material complexity.^47^ Light-based printing provides higher resolution but relies on photopolymerizable bioinks and requires careful control of photo-induced cytotoxicity.^48^

Recent PDAC-oriented studies illustrate how bioprinting can generate structured tumor models and spatially organized co-cultures. Using extrusion-based bioprinting, Godier et al. fabricated a PDAC cancer-stroma construct by co-printing Panc-1 cells and CAFs within an alginate/gelatin hydrogel. Cells remained viable and self-organized into aggregates with heterogeneous cellular composition, highlighting the accessibility of extrusion printing for reproducible multicellular PDAC model building.^46^ In parallel, Monteiro et al. leveraged embedded bioprinting with a GelMA-HAMA ECM-mimetic bioink to fabricate tumor-scale (∼6 mm) PDAC cancer-stroma constructs with engineered spatial compartmentalization, enabling rapid production and drug-dependent gemcitabine testing; importantly, the stromal compartment increased drug resistance relative to monotypic counterparts, underscoring the functional importance of spatial organization and HA-containing matrices in desmoplastic PDAC modeling.^26^

However, a fundamental limitation of extrusion-based bioprinting is the resolution-viability trade-off: achieving higher spatial resolution requires narrower nozzles, which exponentially increases the shear stress experienced by the PDOs and subsequently reduces cell viability. To break this bottleneck, tomographic volumetric bioprinting (VBP) has emerged as a high-speed alternative. By utilizing light projections to induce polymerization of the entire 3D volume simultaneously, VBP can fabricate complex PDAC models—including intricate acinar and ductal structures—in under 60 s, maintaining cell viability above 90% and preserving long-term functionality.^49^ Furthermore, replicating the “desmoplastic barrier” remains a central challenge. Conventional bioinks often fail to mimic the extreme stiffness of PDAC stroma, which can account for over 90% of tumor volume. Recent studies have addressed this limitation by utilizing Collagen -GelMA-Alginate hybrid inks, in which alginate serves as the primary tool for stiffness adjustment, increasing model hardness through ionic cross-linking to simulate the characteristic high physical pressure of PDAC. Simultaneously, collagen I provides the necessary biochemical cues to induce a desmoplastic-like response, which is essential for paclitaxel resistance.^50^

Remaining challenges include balancing print fidelity with cell health (shear/thermal stress), minimizing phototoxicity in light-based approaches, and overcoming diffusion limits in mm-scale constructs.^47^ Future progress will likely hinge on standardizing bioink rheology and post-print mechanics reporting, while integrating perfusion or vascular interfaces to overcome diffusion limits in tumor-scale constructs. In practice, bioprinting often demands specialized equipment, careful matching between printing modality and bioink properties to preserve viability and architecture, which can limit accessibility and inter-lab reproducibility.

### Self-assembly for PDO formation

As a complementary, less deterministic route to bioprinting, self-assembly trades architectural precision for native cell behavior and broader scalability. There are two related but distinct ways of “self-assembly” in PDAC PDO engineering, utilizing the intrinsic aggregating tendencies of biomaterials or cells. First, supramolecular biomaterial self-assembly utilizes peptides to co-assemble into the ECM. Osuna et al. developed a tunable *ex vivo* platform based on 3D co-assembly of peptide amphiphiles (PAs) with custom ECM components, which maintained patient-specific transcriptional profiles and cancer stem cell functionality, and better reproduced patient-specific *in vivo* drug responses compared with conventional PDO models.^36^ In addition, a PA-E3Y self-assembling hydrogel system demonstrated that increasing matrix stiffness induced epithelial-to-mesenchymal transition and fibrosis marker upregulation, enriched cancer stem cells, and enhanced invasiveness and chemoresistance.^35^ Second, scaffold-free self-assembly refers to the spontaneous compaction of tumor and stromal cells without the addition of exogenous ECM. For example, Ware et al. reported a stroma-rich PDAC spheroid model by incorporating pancreatic stellate cells, promoting a dense, collagenous stromal compartment and reduced gemcitabine efficacy.^51^ Furthermore, patient-derived tumor-like cell clusters were described as self-assembled and proliferative clusters containing primary epithelial, fibroblast, and immune cells, shortening the turnaround time for functional drug testing.^52^ Recently, integrin-targeted, matrix minimal strategies have blurred the boundary between scaffold-free and matrix-based cultures by substituting key ECM-derived adhesion signals. A single-chain β1-integrin activator markedly increased organoid yield and supported growth in purified collagen I hydrogels in multiple human epithelial organoids.^53^ Taken together, self-assembly strategies offer a unique balance between formation efficiency and reproducibility. Their primary advantage lies in fostering native cell-cell and cell-matrix interactions in a highly defined, or even scaffold-free, manner, significantly reducing the batch-to-batch variability and xenogenic risks associated with natural extracts. However, compared to 3D bioprinting, self-assembly relies on stochastic cellular or molecular interactions, offering less precise control over the macroscopic spatial organization and defined tissue boundaries. Future efforts may focus on hybrid strategies that offer more programmed morphological control throughout the self-assembly process.

In summary, biofabrication technologies have improved PDO construction, balancing structural complexity and standardization by enabling the automatic, precise positioning of PDAC cells, stromal components, and engineered matrices. Yet, achieving true physiological relevance requires more than structural accuracy; culture success and phenotype are jointly determined by matrix, soluble niche factors, and physical culture parameters. Early PDAC PDO protocols rely on multi-factor media that include EGF, Wnt3a, R-spondin1, and so forth. to sustain expansion.^54^ Later protocol refinements demonstrated that parallel or tailored media conditions—with or without Wnt niche signals—influence organoid growth efficiency and mitigate selection biases linked to Wnt dependency and transcriptional subtype.^55^^,^^56^^,^^57^ Finally, microenvironmental oxygen tension during establishment can shape captured PDAC phenotypes, with hypoxic establishment enriching basal-like features and underscoring oxygen as an additional parameter for protocol refinement and reproducibility.^58^^,^^59^ Collectively, these advances motivate a “matrix + spatial architecture + media + physical parameters” co-standardization mindset, providing a practical bridge from ECM engineering to multi-component cultivation platforms.

## Advanced culture systems for physiological fidelity

### Co-culture systems for tumor-stroma-immune interactions

**Figure 2:**
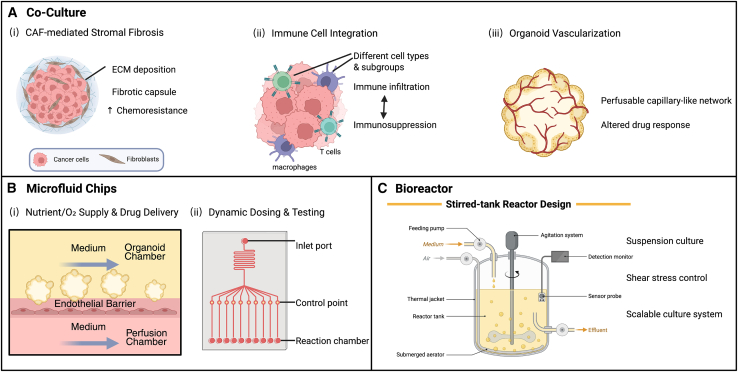
Streamlined bioengineering platforms for PDAC organoid culture (A) Co-culture modules—CAFs induce stromal fibrosis; adding immune cells captures infiltration and tumor-driven suppression; endothelial cells establish perfusable microvessels. (B) Microfluidic chips—endothelial-barrier designs maintain nutrient/O_2_ flow and drug delivery, while multiplexed inlet manifolds enable programmable, high-throughput dosing. (C) Bioreactors—stirred-tank suspension systems scale organoid yield with controlled shear and real-time monitoring. *Created in BioRender*.

Traditional pancreatic tumor organoids, focused on epithelial cell structures, can mimic the structural and genetic characteristics of primary pancreatic cancer lesions but struggle to replicate the tumor’s stromal components. Interactions between **stromal cells** and tumor cells are vital for understanding the biological behavior of pancreatic tumors and predicting their drug sensitivity. Co-culturing organoid models with non-tumor cell components, such as immune cells or fibroblasts, to simulate tumor-stroma interactions and the tumor immune microenvironment offers a powerful research tool to support pancreatic cancer drug screening, personalized treatment, and investigations into the mechanisms of the TME (Figure 2).^60^^,^^61^ We summarize representative co-culture modules later in discussion.

### Fibroblast co-cultures: modeling desmoplasia-driven drug tolerance

Pancreatic cancer, marked by extensive fibrosis, features a dense stroma where **cancer-associated fibroblasts (CAFs)**, a key component of the TME, actively contribute to processes such as tumor progression and metastasis. CAFs in pancreatic cancer secrete tumor growth factors and play key roles in suppressing immune cell activation and regulating tumor cell metabolism.^62^^,^^63^ CAF heterogeneity is functionally important: myofibroblastic and inflammatory CAF subtypes (myCAF and iCAF) exert distinct effects on cytokine signaling, ECM deposition, and therapy response.^64^ Co-culturing pancreatic tumor organoids with CAFs enables more precise tumor characterization and enhances the accuracy of drug sensitivity predictions for anti-tumor therapies.^65^^,^^66^ Jang et al. employed a multilayer spheroid assembly: PDAC cancer cells were aggregated and then encased within layers of patient-derived fibroblasts to form a miniature tumor nodule surrounded by dense fibrosis. The fibrosis-encapsulated tumoroids grew faster and were more resistant to chemotherapy than cancer-cell-only organoids, mirroring the drug resistance conferred by stromal barriers in PDAC.^67^ Single-cell transcriptome sequencing analysis of pancreatic cancer organoids and their matched CAFs revealed patient-specific CAF clustering. Compared to individually cultured CAFs, co-cultured CAFs displayed greater transcriptomic heterogeneity, highlighting the influence of intercellular signaling within the TME.^65^ To better capture CAF diversity and more standardized stromal inputs, Takeuchi et al. incorporated human iPSC-derived stromal cells to generate pancreatic cancer organoids containing heterogeneous CAF states, providing a tractable route to model CAF-driven phenotypes under more controllable conditions.^68^ Moreover, as a poorly vascularized tumor, pancreatic cancer’s hypoxia response mechanisms represent a key focus of TME research. Hypoxic conditions enhance iCAFs, primarily by stabilizing the HIF-1α protein, which plays a central role in this process. In the co-culture system of pancreatic cancer organoids and CAFs, hypoxia-induced HIF-1α stimulates CAFs to secrete increased levels of pro-inflammatory factors, further reinforcing the drug resistance of tumor cells.^69^

### Myeloid co-cultures: linking innate immunosuppression to therapy response

Establishing a co-culture system for pancreatic cancer organoids and immune cells is a crucial approach for investigating the interaction networks within the pancreatic cancer microenvironment and mechanisms of drug resistance. Tumor-associated macrophages (TAMs) and **Myeloid-Derived Suppressor Cells (MDSCs)** can inhibit the function of T cells and other immune cells through various mechanisms, thereby playing a critical role in tumor immune evasion and the inhibition of anti-tumor immune responses.^70^ To simulate the immunosuppressive TME in pancreatic cancer, Zavros’s team developed a co-culture system with pancreatic cancer organoids, MDSCs, and tumor-reactive T cells.^71^ The study demonstrated that MDSCs suppress PD-1 inhibitor-induced organoid apoptosis. A macrophage-organoid co-culture model in PDAC demonstrated macrophage-associated gemcitabine resistance and enabled screening strategies to counteract the resistance, supporting the value of explicitly modeling macrophage-tumor interactions when interpreting drug response *in vitro*.^72^ More recently, PDAC PDOs fused with iPSC-derived macrophages have recapitulated five TAM subtypes *in vitro* and shown how TAM diversity correlates with cancer cell survival and with other TME features within PDO constructs.^73^ A recurrent limitation is that myeloid phenotypes are highly sensitive to medium composition and matrix cues, underscoring the need to report culture conditions and benchmark myeloid state with tumor readouts.

### Immune effector co-cultures: functional killing assays and immunotherapy testing

Immune effector-PDO co-cultures are increasingly used for immunotherapy testing and mechanistic investigation, but they require careful design because immune cell survival/activation programs are often incompatible with standard PDO media. Short-term assays typically add activated PBMCs/T cells/NK cells onto established PDOs to quantify infiltration and killing using time-lapse imaging, cytokine release, and flow-based activation markers. James et al. demonstrated, through the co-culture of PDAC organoids and immune cells, that tumor organoids secrete chemokines that induce the migration of effector T lymphocytes to the periphery of the organoids.^74^ Importantly, recent work demonstrates that PDO co-cultures can be extended from phenotypic immune readouts to antigen-specific development of immunotherapies. Ely et al. used PDAC PDOs to enrich tumor cells and discover immunogenic HLA peptides by immunopeptidomics. They further manufactured TCR-T cells targeting the immunogenic HLA peptides discovered by highly sensitive screening. By transducing PDAC PDOs with eGFP-firefly luciferase reporters, they established quantitative PDO co-culture assays with antigen-specific TCR-T cells and measured cytokine release, cytotoxicity, and activation markers, demonstrating an end-to-end workflow from antigen discovery to functional killing assays based on the PDAC PDO model.^75^ Despite these successes, functional immune-organoid co-cultures suffer from compatibility limitations. First, standard PDO media rely on components such as A83-01 (a TGF-β inhibitor) and nicotinamide, which can disrupt T cell polarization and directly suppress NK cell cytotoxicity, forcing researchers into narrow experimental windows.^76^ Second, dense artificial extracellular matrices (ECMs) and the characteristic desmoplastic stroma of PDAC physically restrict immune cell infiltration, shielding tumor cells from effector contact.^77^ Finally, utilizing allogeneic immune cells introduces severe HLA mismatches that trigger nonspecific, antigen-independent “background killing,” while autologous cell expansion remains technically challenging and time-consuming for real-time clinical utility.^78^ Further efforts have been made toward autologous tissue-resident immune cells in PDO models.^79^

### Vascularized co-cultures: drug gradients and transport barriers

Endothelial cells form the lining of blood vessels and are essential for creating a perfusable network to deliver nutrients and drugs. PDAC is known for sparse vessels. Incorporating endothelial cells into PDO cultures aims to generate vessels to better investigate transport and tumor-vasculature interactions. Vascularized co-culture technology for pancreatic cancer organoids represents a significant focus and challenge in the field of organoid research. Integrating vascular networks into organoid co-culture systems has demonstrated significant value in drug screening. Benjamin’s team developed a three-dimensional vascularized pancreatic cancer organoid culture system co-cultured with CAFs and vascular endothelial cells. Compared to traditional drug sensitivity tests of pancreatic cancer tumor organoids, vascularized culture systems simulate authentic tumor microvascular structures, providing tumor cells with a more complex and dynamic growth environment, thereby affecting drug distribution and drug sensitivity of cells.^80^ Similarly, Takeuchi et al. created a fused pancreatic cancer organoid (FPCO) by co-culturing patient-derived PDAC cells with mesenchymal and vascular endothelial cells derived from human iPSCs, successfully recapitulating the TME *ex vivo* and demonstrating different drug response profiles based on the organoid’s state.^68^ However, a remaining limitation is the difficulty in achieving true *in vitro* anastomosis between the organoid’s internal capillary networks and external fluidic channels. Without functional active perfusion, these structurally impressive vascular networks act as isolated dead ends. Furthermore, the absence of fluidic shear stress stalls endothelial maturation. Therefore, advancing from static co-cultures to dynamically perfused microfluidic platforms is essential for accurate physiological modeling.^81^

Taken together, co-culture systems provide a direct route to restore cellular heterogeneity of PDAC TME into PDO platforms, but their translational utility depends on standardized reporting and quantitative readouts. Because many of these interactions are governed by spatial gradients and dynamic perfusion, the next level of control is often achieved by microfluidic platforms.

### Microfluidics platforms for dynamic TME simulation

Static co-cultures stop short of replicating gradient and perfusion cues. Microfluidic technology replicates the vascular system by creating microfluidic channels and chambers, enabling precise control of culture conditions (such as nutrient, oxygen, and drug delivery).^82^ Goluba et al. created a PDMS-free microfluidic PDAC chip that includes a functional endothelial barrier, where PDOs are cultured as an adherent layer in one channel while a parallel channel is lined with primary human umbilical vein endothelial cells, enabling cultivation for over 50 days and continuous monitoring of tumor growth and health via analysis of the effluent media.^83^ In addition, low-oxygen culture technology, a branch of microfluidic technology, controls oxygen delivery to maintain low-oxygen conditions for tumor organoid cultures. This approach simulates the hypoxic TME of pancreatic cancer, thereby enabling the study of mechanisms related to hypoxic response, tumor invasion, and chemoresistance in pancreatic cancer.^84^

Additionally, microfluidic technology can facilitate the development of a high-throughput drug screening platform for pancreatic cancer organoids. Deipenbrock et al. developed a fit-for-purpose PDAC chip with perfusable channels for drugs and immune cells, highlighting how dynamic dosing can be implemented *in vitro*.^85^ Kramer’s research team developed a novel high-throughput drug screening platform for pancreatic cancer organoids by integrating microfluidic technology with a 3D culture system. They utilized microfluidic chips to facilitate high-throughput drug screening experiments and toxicity assays, demonstrating compatibility with pancreatic cancer tumor organoids.^86^ Schuster’s research team further developed an automated high-throughput microfluidic 3D organoid culture and analysis system, providing integrated organoid cultivation and drug testing capabilities.^82^ By performing single-drug, combination, and sequential screenings on pancreatic tumor organoids, they confirmed the efficacy of the microfluidic high-throughput system in applications involving pancreatic PDOs.^82^ Choi’s research team used microfluidic devices to evaluate the efficacy of treatment strategies targeting tumor organoids derived from pancreatic cancer biopsy samples. Within this system, tumor organoids exhibit phenotypic and genotypic characteristics comparable to those cultured using traditional Matrigel, while offering advantages in morphological consistency and reduced cell number requirements.^87^ Furthermore, this microfluidic organoid culture system can be utilized to advance the sensitivity assessments of pancreatic cancer organoids to chemotherapy and immunotherapy, significantly enhancing the clinical utility of integrating microfluidics with organoid quantification.^87^

### Bioreactors and suspension cultures for organoid scale-up

Cell suspension and bioreactor technologies complement microfluidic platforms by enabling rapid expansion of PDOs, allowing shorter clinical decision turnaround in large-cohort screening, especially for slow-growing PDAC organoids. Dynamic suspension cell culture technology enables the three-dimensional cultivation of organoid spheroids under controlled shear stress conditions. By utilizing the laminar hydrodynamics module of bioreactors to achieve dynamic cell suspension, this method maintains the morphological characteristics of tumor cells, promotes the formation of intercellular connections, and simulates cell-cell interactions within the *in vivo* environment.^88^ Additionally, it reduces DNA damage and genetic drift associated with traditional culture processes. Moreover, the 3D suspended bioreactor system enables large-scale cultivation of pancreatic progenitor cells through the staggered provision of growth factors and small-molecule compounds. It induces their sequential differentiation and assembly into pancreatic cell clusters with insulin-secreting functions, offering new biotechnological methods for islet transplantation therapy.^89^ Cell suspension culture and bioreactor technologies hold significant potential applications in the cultivation of pancreatic cancer tumor organoids and in pancreatic regenerative medicine research. They provide new tools and methods for studying oncological mechanisms and developing personalized therapies.

To sum up, advanced culturing strategies offer better environmental control and incorporate more complex interactions that shed light on systemic disease pathology. Aside from complex interactions, PDAC PDOs can capture heterogeneity within tumor cells: Single-cell transcriptome profiling has shown that “classical” and “basal-like” malignant cells can coexist within individual PDO lines and that distinct malignant cell states are connected by a differentiation hierarchy.^10^ Besides, PDOs are not static during extended culture—single-cell whole-genome sequencing suggests that clonal proportions can drift with passaging.^90^ Bioengineering variables (e.g., stiffness and oxygenation) can also bias phenotypes and potentially subpopulation representation over time.^15^^,^^84^ These considerations further motivate the need for quantitative, longitudinal monitoring tools, discussed in the next section.

## Quantitative monitoring pipelines for observability

### Imaging-based structural and dynamic monitoring of PDAC organoids

Beyond simple 2D brightfield projections, advancing organoid monitoring requires capturing dynamic, 3D morphological changes without disrupting the culture. Recent studies have leveraged advanced imaging and image analysis to monitor PDAC organoid growth, phenotypic changes, and treatment responses in unprecedented detail (Table 2). The tools summarized in Table 2 constitute a multiscale observability stack for PDAC organoids: Structural imaging defines what the culture looks like, AI-assisted longitudinal analysis defines how it evolves, live reporters define why it responds, and metabolic readouts define its functional state. To start with, Cutrona et al. conducted a high-content imaging screen on a cohort of PDAC organoids to validate a novel drug target. This image-based approach revealed that organoids with high CYP3A5 (cytochrome P450 3A5, a drug-metabolizing enzyme) activity were sensitized to chemotherapy when treated with the selective CYP3A5 inhibitor.^92^ Methodologically, the assay miniaturizes ECM-embedded PDOs into multiwell plates, applies automated spinning-disk imaging after phalloidin/DRAQ5 staining, and segments each organoid to output area and nuclear count, which together resolve intra- and inter-patient heterogeneity at the single-object level. This transformed drug response into a CYP3A5-dependent phenotypic profile, highlighting the restoration of cisplatin chemo-vulnerability in a CYP3A5-high subset. Aside from live-cell imaging, embedded tissue imaging provides in-depth topology details. A recent study employed three-dimensional imaging (FLIP-IT, fluorescence light-sheet microscopic imaging of paraffin-embedded or intact tissue) to examine formalin-fixed human pancreatic ducts. This study identified for the first time a novel ΔNp63-expressing (ΔNp63 is an N-terminally truncated isoform of TP63 and a commonly used basal-cell marker) basal cell type and investigated its potential role in the pathogenesis of pancreatic diseases, including PDAC. Optical clearing combined with light-sheet acquisition resolved ΔNp63+ basal cells along the basal membrane of medium-to-large ducts at single-cell resolution and showed them forming multilayer dome-like structures in chronic pancreatitis—a niche that scRNA-seq missed and 2D sections could not localize. The trade-off is throughput, as cleared light-sheet workflows are inherently endpoint and tissue-oriented. FLIP-IT is therefore more of a deep-tissue spatial-discovery pipeline than a PDO drug-response assay.^93^ Furthermore, to facilitate high-content data analysis, recent advancements have introduced fully digitalized pipelines such as the AI-based multilevel segmentation tool 3DCellScope, designed for high-speed 3D analysis of organoid structures. Applied to primary PDAC and colorectal cancer organoids, this digitalized approach enables precise tracking of cellular topology and spatial modifications under external stress at both the nuclear and whole-organoid scales. Concretely, the pipeline outputs hundreds of nucleus-, neighborhood-, and organoid-level descriptors, pushing 3D observability beyond size and count toward measurable spatial topology.^97^ However, a significant limitation remains: the integration of such high-resolution, AI-driven 3D imaging pipelines demands massive computational resources and remains technically challenging to integrate with microfluidic chips due to hardware incompatibilities and optical transparency issues. Taken together, structural imaging transforms PDAC organoid cultures into measurable object distributions linked with phenotypes, but still requires computational pipelines that segment organoids and track changes over time and predict which signaling drives that change—described in the next subsection.

**Table 2:** Advanced monitoring tools for PDAC organoids

Monitoring technology	Subtype/Example	Measurable parameters	Resolution and scale	Advantages	Limitations	Refs
Microscopic imaging	live-cell imaging systems	cellular activities, morphology, growth, drug response, intracellular signaling (e.g., ERK/AMPK)	single-cell; long-term (hours-days)	dynamic monitoring of cellular activities, growth, drug response, signaling; temporal resolution	lower throughput vs endpoint assays, potential phototoxicity	^91^
	high-content imaging	multi-parametric viability, morphology, drug response (e.g., based on specific markers such as CYP3A5 activity)	subcellular resolution; endpoint or time-course; high-throughput	high-throughput screening; quantitative, multi-parametric analysis	limited dynamic tracking; requires complex image analysis pipelines	^92^
	FLIP-IT	complex 3D structures, spatial organization (e.g., ducts, cell types)	subcellular resolution; organoid/tissue scale	visualizes complex 3D structures and spatial organization	requires optical clearing and advanced imaging platforms; lower throughput; limited penetration in dense tissues	^93^
AI-assisted image analysis	MOrgAna	morphology, fluorescence data, segmentation	organoid level	quantitative, automated morphology and fluorescence analysis; open-source	primarily for 2D imaging; limited capability for highly complex or dense organoids	^94^
	OrganoID	organoid morphology (area, shape, eccentricity), growth kinetics	organoid level; time-lapse	automated tracking of individual organoids over time; open-source	focuses on morphological data	^95^
	OrganoIDNet	organoid morphology, fragmentation, lumen darkening, apoptosis, and drug response dynamics	organoid level; time-lapse	analyzes PDAC organoid dynamics, including co-cultures; early drug/immune response indication; open-source	trained on brightfield images; limited generalizability	^96^
	3DCellScope	3D cellular topology, multilevel spatial modifications, nuclear/cytoplasmic morphological metrics	subcellular resolution, spatial topology	high-speed, quantitative digitalization of complex spatial relationships and dynamic morphological responses to stress	massive computational infrastructure	^97^
Metabolic monitoring	optical metabolic imaging (OMI)	cellular metabolism (e.g., NAD(P)H/FAD autofluorescence, OMI index)	cellular level	label-free metabolic readout; distinguishes subpopulations; longitudinal (hours-days)	requires two-photon FLIM system; low throughput; limited imaging depth	^98^

Building on the overview in Table 2, the next subsections highlight two representative monitoring axes: AI-assisted image analysis and metabolic/microsensor readouts, which complement each other for scalable phenotyping.

### AI-assisted image analysis of tumor organoids

Artificial intelligence technology has achieved significant advancements in the recognition and analysis of image data, facilitating better understanding and explanation of morphological changes. Utilizing image segmentation and time-lapse tracking models, researchers could more accurately capture and analyze morphological and microstructural changes in organoids without relying on complex imaging instruments.^99^ For instance, the Python-based MOrgAna software utilizes machine learning for image segmentation, allowing more precise visualization and quantification of morphological and fluorescence data in tumor organoids.^94^ The OrganoID deep learning platform is capable of automatically recognizing organoids in light field and phase-contrast microscopy experiments and can label and continuously track individual organoids.^95^ The OrganoID artificial intelligence image recognition system employs high-precision tracking and analysis of morphological changes in tumor organoids subjected to drug treatments, providing convenient and reliable technical tools for high-throughput drug sensitivity screening of pancreatic cancer organoids.^95^ In 2024, Ferreira et al. introduced OrganoIDNet, a deep learning tool specifically designed to analyze time-lapse images of PDAC organoids, including those co-cultured with immune cells.^96^ The researchers generated a large dataset of phase-contrast images of human and murine PDAC organoids and trained a neural network to monitor organoids based on morphological changes such as fragmentation or darkening, providing an earlier indication of drug response and evaluation of immunotherapy. Specifically, OrganoIDNet quantifies count, area, eccentricity, size class, and a per-organoid health-status label from phase-contrast time-lapse images of PDAC organoid-PBMC co-cultures, distinguishing fragmentation and immune-mediated death from organoid fusion under gemcitabine and HLA-matched atezolizumab. The companion OrganoIDNetData benchmark (33,906 expert-annotated organoids with retrained Cellpose and StarDist) underscores that AI-assisted PDO monitoring is only as reliable as the dataset and train-validation pipeline behind it.^96^ Moreover, a 2025 study by Tsukamoto et al. applied quantitative live-cell imaging to PDAC PDOs to dissect intracellular signaling dynamics during organoid growth. Using ERK and AMPK FRET biosensors and multiphoton tile-scan imaging in ten PDOs, they showed that PDAC growth is phase-dependent (ERK-dominated early, AMPK-dominated later); accordingly, MEK and autophagy inhibitors targeted different organoid subpopulations and only their combination broadly suppressed heterogeneous PDOs. This explains why endpoint viability assays can mask stage-specific resistant subpopulations, though the FRET workflow remains too demanding for routine high-throughput use.^91^ Complementarily, spatially resolved multi-omics profiling (scRNA-seq, spatial transcriptomics, multiplex immunohistochemistry, and mass cytometry) has provided a fine map of immune dysfunction in treatment-naïve PDAC.^100^ These advancements contribute to the practicality and reliability of PDAC organoid models in drug development, disease simulation, and personalized medicine.

### Metabolic and microsensor-based organoid monitoring

Real-time monitoring technology for pancreatic cancer organoids, based on metabolic analysis and biosensing technology, facilitates researchers in tracking and analyzing tumor cell behavior, drug responses, and disease progression. Walsh’s research team developed Optical Metabolic Imaging (OMI) technology to assess cellular metabolic changes induced by anticancer therapeutics. The technology utilizes quantification of the OMI index as a biomarker for drug responses in pancreatic cancer organoids and distinguishes among cell subpopulations based on their differential drug responses. It provides longitudinal drug response data, distinguishing responders from non-responders and thereby reflecting differential treatment effects across cell populations. Mechanistically, OMI derives a single index from endogenous NAD(P)H and FAD autofluorescence in living organoids; in murine and human PDAC PDOs, OMI shifts identified responding and non-responding subpopulations and correlated with Ki67 and cleaved-caspase-3 staining—a nondestructive functional readout that endpoint dye assays cannot resolve, but one that requires two-photon/FLIM instrumentation and remains low-throughput.^98^ In the future, real-time monitoring of pancreatic cancer organoids through integrated microsensors can provide dynamic data, assisting researchers in understanding tumor behavior at the cellular level, evaluating new drug efficacy, and developing more effective personalized treatment strategies. On-chip lens-free imaging has proven effective in cell lines and could potentially be integrated for organoid observation and analysis.^101^^,^^102^ Electrochemical impedance spectroscopy has shown promise in cell adherence and viability monitoring; however, its application in organoid monitoring remains to be fully explored.^103^ These microsensor- and impedance-based readouts remain to be validated for PDAC PDO monitoring, as most evidence comes from cell-line or single-cell systems, and sensor surfaces and electrical measurements may themselves perturb cell adhesion and drug response. Co-validation against imaging and viability benchmarks is still needed before translational use.

## Concluding remarks and future perspectives

The widespread application of bioengineering techniques has enhanced the overall PDAC organoid pipeline for construction, culture, and monitoring, playing an important and expanding role in simulating complex TME, evaluating drug responses, and exploring cancer biological mechanisms. From a precision-medicine perspective, PDAC PDOs are increasingly explored for functional drug-response testing; notably, microfluidic organoid cultures derived from pancreatic cancer biopsies enable personalized evaluation of chemotherapy and immunotherapy using limited starting material.^87^ Based on bioengineered PDO models, the development of targeted and personalized therapies for PDAC could be accelerated, as high-content organoid screening can capture patient-specific sensitivity and chemoresistance phenotypes in experimental settings.^92^ Beyond chemotherapy, immune-enabled co-cultures further extend functional precision testing toward immunotherapies, ranging from immune-cell infiltration/killing assays to antigen-discovery to TCR-T cell validation workflows.^75^ However, clinical integration remains in early stages; prospective outcome validation and workflow standardization—assay QC, turnaround time, success rate, and inter-laboratory reproducibility—remain key bottlenecks. In addition, key challenges for implementing bioengineered PDO platforms include standardizing and transparently reporting design parameters (matrix composition/mechanics and co-culture cell-state composition), mitigating diffusion limitations through perfusion/vascular interfaces in more complex constructs, and balancing model fidelity with throughput, cost, and accessibility for multi-center reproducibility. Looking forward, the further integration of advanced bioengineering technologies such as microsensors and AI-driven multimodal data analysis will continue to drive significant advancements and breakthroughs in the investigation of pancreatic cancer through bioengineered tumor organoid models.

### Outstanding questions box

1. How can we achieve robust, scalable, and cost-effective standardization of bioengineered matrix production and organoid culture protocols to ensure inter-laboratory reproducibility and facilitate clinical adoption?
2. What bioengineering strategies can effectively incorporate the full complexity of the tumor microenvironments, including diverse immune cell populations (e.g., T cells, MDSCs, and macrophages), neuronal influences, and dynamic extracellular matrix remodeling?
3. How can stable, perfusable, and physiologically relevant vascular networks be reliably integrated within pancreatic ductal adenocarcinoma (PDAC) organoid models to accurately mimic *in vivo* drug delivery and tumor vascularization?
4. How can we improve the long-term culture of bioengineered PDAC organoids while maintaining their genetic and phenotypic stability, as well as their representation of intra-tumoral heterogeneity, clonal stability, and phenotypic evolution during passaging?
5. What are the most effective methods for integrating real-time, non-invasive biosensing technologies within organoid platforms to continuously monitor metabolic activity, signaling pathways, and drug responses?
6. How can multiparametric data (imaging, functional, omics, including spatial-omics, and biosensing data) be integrated using AI to build truly predictive models of patient-specific drug efficacy?
7. How can engineered PDAC organoid functional assays be prospectively validated and standardized to support treatment decision-making within clinically actionable turnaround times?

**Glossary**

**Cancer-associated fibroblasts (CAFs)**: A major cellular component of the tumor stroma (microenvironment) in many cancers, including PDAC. These activated fibroblasts interact with cancer cells and can promote tumor growth, invasion, metastasis, drug resistance, and immune suppression.
**Extracellular matrix (ECM)**: The non-cellular network of proteins (such as collagen, laminin, and fibronectin) and polysaccharides that surrounds cells in tissues, providing structural support and regulating cell adhesion, migration, proliferation, and differentiation.
**Gelatin methacryloyl (GelMA)**: A semi-synthetic hydrogel derived from gelatin modified with methacryloyl groups, which can be rapidly crosslinked using photoinitiators and UV or visible light to form stable hydrogels with tunable mechanical properties.
**Heterogeneity**: Variation among cells within a single tumor or between tumors from different patients. Heterogeneity can be genetic, epigenetic, or phenotypic and impacts tumor behavior and treatment response.
**Hydrogel**: A three-dimensional network of hydrophilic polymers that can absorb large amounts of water while maintaining its structure. Used extensively in bioengineering due to their biocompatibility and tunable properties.
**Matrigel**: A commercially available, protein-rich basement membrane extract derived from Engelbreth-Holm-Swarm (EHS) mouse sarcoma cells. Commonly used as a scaffold for 3D cell culture.
**Microfluidics**: The science and technology of manipulating and controlling fluids, typically in the sub-milliliter range, within networks of micro-channels.
**Myeloid-derived suppressor cells (MDSCs)**: A heterogeneous population of immature myeloid cells that accumulate in tumor microenvironments and exert immunosuppressive functions.
**Organoid:** Three-dimensional, miniature organ-like structures grown from tumor cells or stem cells that replicate much of the architecture and functionality of actual tumors or organs. Used to model diseases and test drug responses.
**Patient-derived organoid (PDO)**: Organoid generated directly from a patient’s tissue sample (e.g., tumor biopsy) that self-organizes to recapitulate key structural, functional, and genetic characteristics of the original tissue or tumor *in vitro*.
**Patient-derived tumor xenograft (PDTX)**: An animal model (typically immunodeficient mice) where fragments of a patient’s tumor are surgically implanted and grown.
**Pancreatic ductal adenocarcinoma (PDAC)**: The most common type of pancreatic cancer, arising from the cells lining the pancreatic ducts. It is characterized by aggressive growth, early metastasis, dense fibrotic stroma, and poor prognosis.
**Polyethylene glycol (PEG)**: A biocompatible, versatile synthetic polymer often used to create hydrogels for biomedical applications. PEG hydrogels can be engineered with specific properties (e.g., stiffness and degradability) and functionalized with bioactive molecules for controlled organoid culture.
**Stromal Cells**: Non-cancerous cells within the tumor microenvironment, including fibroblasts (such as CAFs), immune cells, endothelial cells (lining blood vessels), and pericytes, which significantly influence tumor behavior.
**Tumor microenvironment (TME)**: The complex ecosystem surrounding a tumor, comprising the ECM, stromal cells (fibroblasts, immune cells, endothelial cells), blood vessels, lymphatics, and signaling molecules. The TME critically influences tumor growth, invasion, metastasis, and response to therapy.

## Acknowledgments

This work was supported by the 10.13039/501100001809National Natural Science Foundation of China (W. Wu, no. 52271254).

## Author contributions

Conceptualization, R.C., B.Y., X.G., and W.W.; literature review, R.C., B.Y., Y.Z., and J.P.; writing – original draft, R.C. and B.Y.; writing – review and editing, R.C., B.Y., Y.Z., J.P., X.G., and W.W.; supervision, X.G. and W.W.; funding acquisition, W.W.

## Declaration of interests

The authors declare no competing interests.

## Declaration of generative AI and AI-assisted technologies in the writing process

During the preparation of this work, the authors used ChatGPT in order to assist with English language editing and readability improvement. After using this tool, the authors reviewed and edited the content as needed and take full responsibility for the content of the publication.
